# Healthy Mom Zone Adaptive Intervention With a Novel Control System and Digital Platform to Manage Gestational Weight Gain in Pregnant Women With Overweight or Obesity: Study Design and Protocol for a Randomized Controlled Trial

**DOI:** 10.2196/66637

**Published:** 2025-03-13

**Authors:** Danielle Symons Downs, Abigail M Pauley, Daniel E Rivera, Jennifer S Savage, Amy M Moore, Danying Shao, Sy-Miin Chow, Constantino Lagoa, Jaimey M Pauli, Owais Khan, Allen Kunselman

**Affiliations:** 1 Department of Kinesiology Pennsylvania State University University Park, PA United States; 2 Department of Obstetrics and Gynecology College of Medicine Pennsylvania State University Hershey, PA United States; 3 School of Engineering of Matter, Transport and Energy Arizona State University Tempe, AZ United States; 4 Department of Nutrition Center for Childhood Obesity Research Pennsylvania State University University Park, PA United States; 5 Institute for Computational and Data Sciences Pennsylvania State University University Park, PA United States; 6 Human Development and Family Studies Quantitative Developmental Systems Methodology Core Pennsylvania State University University Park, PA United States; 7 College of Engineering, School of Electrical Engineering and Computer Science Pennsylvania State University University Park, PA United States; 8 Division of Maternal Fetal Medicine College of Medicine Pennsylvania State University Hershey, PA United States; 9 Department of Public Health Services, Division of Biostatistics and Bioinformatics College of Medicine Pennsylvania State University Hershey, PA United States

**Keywords:** pregnancy, gestational weight gain, physical activity, healthy eating, overweight, obesity, intervention

## Abstract

**Background:**

Regulating gestational weight gain (GWG) in pregnant women with overweight or obesity is difficult, particularly because of the narrow range of recommended GWG for optimal health outcomes. Given that many pregnant women show excessive GWG and considering the lack of a “gold standard” intervention to manage GWG, there is a timely need for effective and efficient approaches to regulate GWG. We have enhanced the Healthy Mom Zone (HMZ) 2.0 intervention with a novel digital platform, automated dosage changes, and personalized strategies to regulate GWG, and our pilot study demonstrated successful recruitment, compliance, and utility of our new control system and digital platform.

**Objective:**

The goal of this paper is to describe the study protocol for a randomized controlled optimization trial to examine the efficacy of the enhanced HMZ 2.0 intervention with the new automated control system and digital platform to regulate GWG and influence secondary maternal and infant outcomes while collecting implementation data to inform future scalability.

**Methods:**

This is an efficacy study using a randomized controlled trial design. HMZ 2.0 is a multidosage, theoretically based, and individually tailored adaptive intervention that is delivered through a novel digital platform with an automated link of participant data to a new model-based predictive control algorithm to predict GWG. Our new control system computes individual dosage changes and produces personalized physical activity (PA) and energy intake (EI) strategies to deliver just-in-time dosage change recommendations to regulate GWG. Participants are 144 pregnant women with overweight or obesity randomized to an intervention (n=72) or attention control (n=72) group, stratified by prepregnancy BMI (<29.9 vs ≥30 kg/m^2^), and they will participate from approximately 8 to 36 weeks of gestation. The sample size is based on GWG (primary outcome) and informed by our feasibility trial showing a 21% reduction in GWG in the intervention group compared to the control group, with 3% dropout. Secondary outcomes include PA, EI, sedentary and sleep behaviors, social cognitive determinants, adverse pregnancy and delivery outcomes, infant birth weight, and implementation outcomes. Analyses will include descriptive statistics, time series and fixed effects meta-analytic approaches, and mixed effects models.

**Results:**

Recruitment started in April 2024, and enrollment will continue through May 2027. The primary (GWG) and secondary (eg, maternal and infant health) outcome results will be analyzed, posted on ClinicalTrials.gov, and published after January 2028.

**Conclusions:**

Examining the efficacy of the novel HMZ 2.0 intervention in terms of GWG and secondary outcomes expands the boundaries of current GWG interventions and has high clinical and public health impact. There is excellent potential to further refine HMZ 2.0 to scale-up use of the novel digital platform by clinicians as an adjunct treatment in prenatal care to regulate GWG in all pregnant women.

**International Registered Report Identifier (IRRID):**

DERR1-10.2196/66637

## Introduction

### Background

High maternal prepregnancy BMI and high gestational weight gain (GWG) elevate the risks for poor pregnancy outcomes (eg, gestational diabetes and hypertension) and fetal outcomes (eg, large for gestational age birth weight) [1-6]. High BMI and GWG may also “program” the child’s metabolism for life [7,8] and increase the future risks for obesity and type 2 diabetes in both mothers and their offspring [1-6]. As such, managing GWG has high clinical and public health significance, and it can improve maternal health and impact the etiology of obesity or diabetes in offspring at a crucial time in the life cycle [1-6,8].

Guidelines from the Institutes of Medicine (IOM) [1] and National Academy of Medicine [2] recommend that the optimal total GWG should be based on a woman’s prepregnancy BMI category (ie, overweight: 6.8-11.3 kg; obese: 5.0-9.1 kg). However, evidence from a meta-analytic review including over 1 million pregnant women found that nearly 50% of women exceeded their recommended goals [9], and this was prevalent among those with normal weight as well as those with overweight or obesity. Furthermore, data from a meta-analysis of almost 200,000 women from 25 international cohort studies in the LifeCycle Project [6] showed the highest risk for adverse outcomes among women with both high BMI and high GWG. Given these concerns as well as the rapidly changing landscape of health care delivery since the COVID-19 pandemic, there is a critical and timely need for scalable approaches to effectively regulate GWG. One such strategy that may reach more pregnant women and reduce the burden on prenatal clinicians who monitor GWG is an automated approach that relies on a digital platform with remote delivery and passive remote data collection to monitor and effectively and efficiently regulate GWG.

### Prior Work

Our team’s prior work successfully constructed energy balance models to predict maternal GWG [10] and infant birth weight [11]. Expanding the work of Thomas et al [12], we built a novel dynamic mathematical model of energy balance and behavior to predict GWG [13,14]. It describes how physical activity (PA) and energy intake (EI) behaviors are influenced by social cognitive determinants (attitude, subjective norm, perceived control, intention, and self-regulation) [15-17] and depicts how components (ie, education, behavior coaching, goal setting, nutrition counseling, and engaging in PA and healthy eating activities) impact PA and EI social cognitive determinants and behaviors to regulate GWG. We also explored how the energy balance model could be extended to explain infant birth weight [11,18].

Prior research has shown that behavioral interventions can impact GWG [19-27] and more specifically that participants who received behavioral intervention components (eg, counseling, guided PA, and prescribed diet) had a lower mean GWG, decreased likelihood of exceeding GWG guidelines, and lower risk for adverse maternal and infant outcomes [20]. There was also evidence for a dose-response relationship whereby intensive interventions with more subject contact were associated with a stronger impact on GWG [20]. Moreover, the findings from qualitative and prospective cohort studies support an intensive approach to managing GWG in pregnant women with overweight or obesity because they may be more likely than women with underweight or normal weight to overestimate the amount of weight they should gain, underreport EI, and have low motivation to engage in PA on their own [28-41]. Taken together, there is ample evidence from the literature for an approach that considers the unique needs of pregnant women with overweight or obesity and personalizes intervention dosages to regulate GWG.

Many GWG interventions use a “one size fits all” approach and are not designed to consider individual variability in how women gain weight over the course of gestation. Our approach adapts personalized dosages for each woman in a way that gives more intensive treatment only to the women who need more assistance to regulate GWG. We piloted a proof-of-concept study [42] and feasibility-initial impact randomized trial [8,10,19,43-45] to examine the impact of the Healthy Mom Zone (HMZ) intervention on GWG. The social cognitive theory–based components [15-17] noted above were designed with the Multiphase Optimization Strategy [46] translational science framework [47-50], and control systems methodology [51-55], with the long-term goal to scale-up use by clinicians as an adjunct treatment to prenatal care in order to regulate GWG. This multiphase approach [46] builds an intervention in a principled manner whereby key constraints expected to impact scalability (eg, implementation feasibility and subject or staff burden) are considered from the start so that the end goal is an optimized (effective and efficient) and scalable intervention that delivers the best possible outcome [46]. Our translational science framework [47-50] guided by the Quality Implementation Framework [49] and Quality Implementation Tool [50] aligns with the paradigm shift in the literature to prospectively examine implementation markers (eg, subject acceptability, dosage exposure, and staff burden) from the start of an intervention to identify and resolve challenges during delivery that impact efficacy and scalability [49,50].

Our feasibility trial that randomized 31 pregnant women with overweight or obesity to the adaptive GWG intervention (delivered in person or remotely) or a control group over the course of pregnancy and used mobile health tools and online surveys to measure study outcomes showed high measurement compliance (85%), low burden (eg, average 1 min per day to complete measures), and low attrition (3%) [19]. The control system driven by decision rules and a woman’s observed GWG informed when to adapt dosages (GWG within goals [1,2], maintain dosage; GWG greater than goals, adapt dosage) [19]. Participants in the intervention group had a 21% lower mean GWG and were more likely to have GWG within goals than controls. Exploratory analyses showed promise for the HMZ intervention to impact secondary outcomes, including PA minutes, active kcal, EI kcal, and PA and EI social cognitive determinants. Furthermore, maternal nighttime awakenings were related to higher GWG [45], and maternal eating behaviors (uncontrolled eating or restraint) were related to GWG and infant birth weight [44].

**Figure 1:**
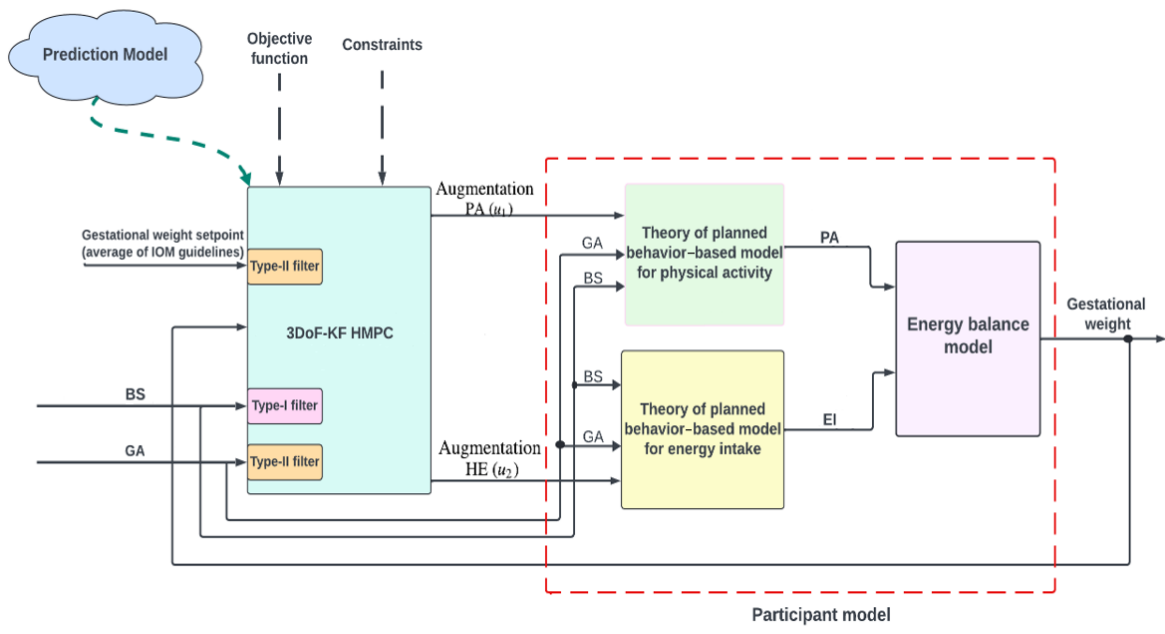
Dynamic model of energy balance and behavior to predict gestational weight gain (GWG). The intervention components are education, counseling, goal-setting, self-monitoring, and physical activity (PA)/healthy eating (HE) active behavior strategies (eg, guided activity sessions and cooking demonstrations). The figure depicts the block diagram of the closed-loop control system framework for managing GWG in pregnant women with overweight or obesity and describes the influence of intervention components, baseline intervention dosage, and gestational age (GA) on GWG. The participant model consists of 2 behavioral models targeting PA and energy intake (EI) behaviors, respectively, and an energy balance model. Outputs from the behavioral models serve as inputs to the energy balance model, which calculates changes in GWG by assessing the difference between EI and energy expenditure. The hybrid model predictive controller (HMPC) uses filtered signals of set point and measured disturbances (baseline intervention dosage and GA). A Type-I filter is used to filter the baseline, represented by a binary signal that indicates the preintervention phase (0) or the intervention phase (1). In contrast, a Type-II filter is used to filter the set point and GA. The HMPC-based optimizer determines a sequence of control actions, referred to as PA and HE dosage augmentations. BS: baseline; IOM: Institutes of Medicine.

Because our long-term goal is to deliver the best possible impact on GWG and maternal or infant outcomes and develop an approach that is scalable for future use in the real world, we used these feasibility findings to make refinements to the intervention content (eg, promoting healthy sleep or eating behaviors), delivery (all content available for remote delivery to ensure scalability), and decision process for evaluating and predicting GWG. More specifically, our initial control system was manually operated (ie, plotted each woman’s weight in individual participant files) and not particularly scalable. We thus improved this system by designing a new model-based control system with a hybrid model predictive control algorithm and our dynamic model of energy balance and behavior (Figure 1) [10,13,56]. This enables projections of within-person GWG over time (even when data are missing at the current time point) and has been found in past studies to yield robust personalized suggestions and accurate inferential results even with missingness [56-68]. We also built an architecture for a novel digital platform that makes it possible to deliver just-in-time recommendations directly to a subject in ways that target a broader array of outcomes and can reach more women. The platform provides a web-based interface equipped with secure user access control that automates the linkage of subject data collected with mHealth tools to the new model-based predictive control system that implements a Control Optimization Trial approach [10,51] consisting of semiphysical system identification and Hybrid Model Predictive Control. The platform displays graphical or numeric summaries of a woman’s past weight, behaviors (eg, PA, EI, and sleep), social cognitive determinants, and other factors; computes optimized dosage changes across multiple maternal variables; and produces a host of personalized PA or EI strategies to regulate GWG. This refined version of the intervention is denoted HMZ 2.0.

In order to activate recruitment, examine participant compliance with the measurement protocol, pilot test the HMZ 2.0 data transfer pipeline, examine intervention session delivery and user acceptability of the digital platform, and conduct simulations for controller-recommended dosage changes, we conducted a 28-day pilot study.

#### HMZ 2.0 Pilot Study Results

We recruited and enrolled 10 pregnant women (mean age: 32.1 years; mean gestational age at enrollment: 14 weeks). Participants completed the HMZ 2.0 preintervention measurement protocol lasting 7 to 9 days (depending on the day of enrollment), completed daily and weekly study measures over a 3-week period while also participating in three 60-minute weekly intervention sessions (content from the first 3 weeks of the HMZ 2.0 baseline intervention) delivered by a trained staff member through the HMZ 2.0 digital platform, and completed the postintervention measurement protocol (7-9 days) and a poststudy brief interview to understand user acceptability. The results of this pilot study are presented below.

We had 59 participant contacts, of which 31 (53%) were assessed for eligibility and 10 (32% of eligible participants) were enrolled in the pilot study. Successful recruitment of participants involved social media (30%), community-based flyers and handouts (eg, campus locations and local events such as farmer’s markets; 30%), word of mouth (20%), and clinic referrals (20%).

Participant compliance with the HMZ 2.0 measurement protocol was excellent, with compliance rates of 88% for the daily or weekly online surveys and MyFitnessPal app, 92% for using the Fitbit Aria Wi-Fi scale each day to assess weight, and 98% for wearing the Fitbit Charge 6 monitor each day to assess PA, sedentary behavior, and sleep behaviors. Moreover, the flow of data through the HMZ 2.0 pipeline was exceptional. Data transfer from devices and Research Electronic Data Capture (REDCap) to the digital platform was smooth, with <2% evidence of technical problems or glitches, and 100% of problems were resolved without issue.

A total of 30 intervention sessions (3 per participant) were delivered, of which 80% (24/30) were delivered with a hybrid approach (in person with the platform) and 20% (6/30) were delivered with a fully remote approach using Zoom and the platform. Attendance and compliance with the sessions were 100%. All the women (10/10, 100%) liked the utility of the HMZ 2.0 digital platform, and 90% (9/10) liked to see their real-time data and set weekly goals with trained study staff. In the poststudy brief interview, 1 woman commented that it was “nice to use [the platform] prior to sessions so I was able to come prepared and ask questions.” Another woman said, “The platform was easy to move from page to page.” A third woman noted, “We covered different topics during the sessions, so I liked being able to go back to the platform and look at the content again.” The participants also provided constructive feedback on the platform and suggested the following refinements: soften the color scheme on the website pages, include a page with session schedule and location information, and include a link to directly email the study staff to reduce the burden of looking through study materials for contact information. The platform was refined to address these concerns.

**Figure 2:**
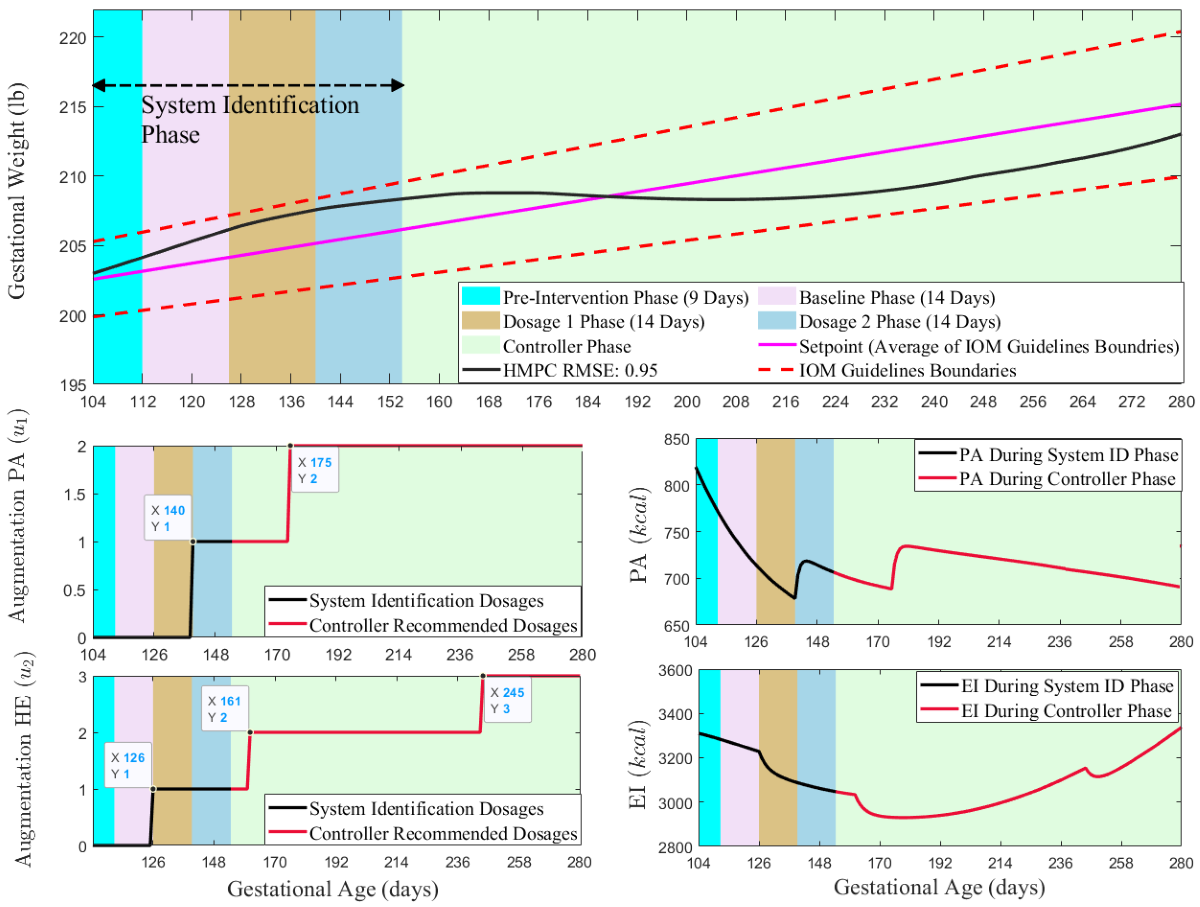
Simulation illustrating the various stages of the control optimization trial implemented in the Healthy Mom Zone (HMZ) 2.0 intervention using an estimated model of an HMZ 1.0 representative participant. EI: energy intake; HE: healthy eating; HMPC: hybrid model predictive controller; IOM: Institutes of Medicine; PA: physical activity; RMSE: root mean square error.

Simulations to illustrate the estimated model are presented in Figure 2, which shows the Control Optimization Trial framework for the recommended dosages within the system identification phase, adhering to the 2-week and 3-week delay. The first panel displays the participant’s weight in pounds (black solid line) and the setpoint (solid magenta line), which indicates the mean of the IOM guidelines (dashed red line) [1]. Different colors are used to represent the various intervention phases. The preintervention phase lasts for 7 to 9 days (9 days in this case), followed by a 2-week baseline period. During both the preintervention and baseline phases, no augmentations or dosages are administered. The first dosage is recommended after 2 weeks in the dosage 1 phase, starting on gestational day 126. The second dosage follows 2 weeks after dosage 1 in the dosage 2 phase. The second panel contains 2 subpanels: one for the manipulated variable augmentation PA and another for PA kcal. The last panel similarly includes subpanels for augmentation healthy eating and EI kcal. The simulation results show that the hybrid model predictive controller effectively manages the participant’s weight within the IOM bounds once the controller is activated. As illustrated, both in the system identification and controller phases, recommendations for PA or healthy eating dosages lead to reduced EI (healthy eating dosage recommended) or increased PA (PA dosage recommended), ultimately facilitating weight reduction through decreased EI and increased PA.

**Figure 3:**
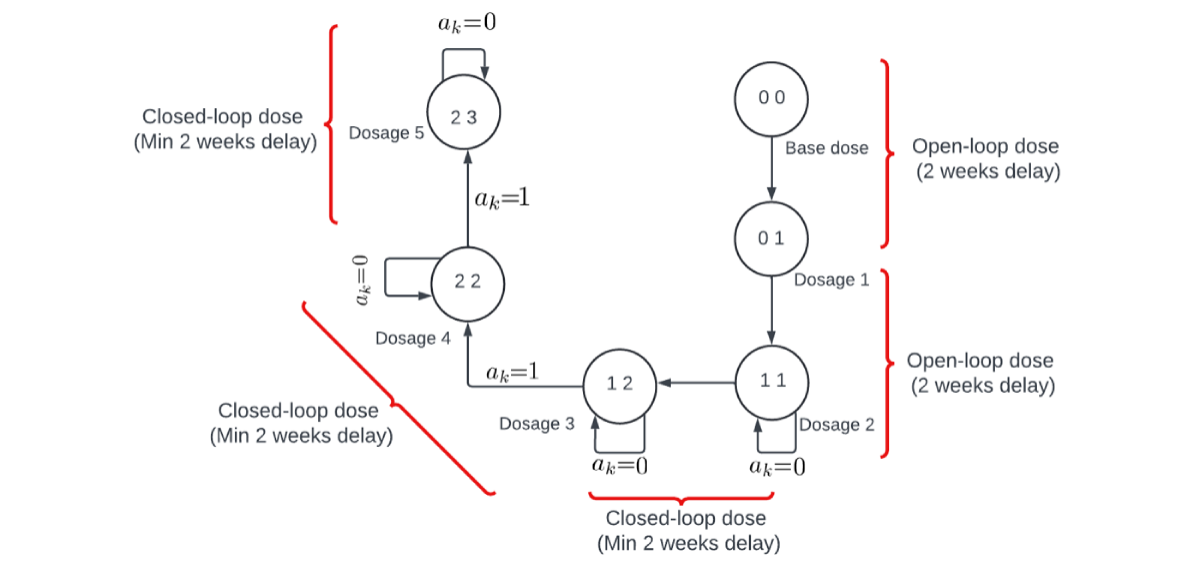
Dosage sequence pattern for the hybrid model predictive controller within the Healthy Mom Zone (HMZ) 2.0 intervention.

After the dosage 2 phase, the controller phase begins, during which the controller recommends further dosages based on predicted participant weight and the logic outlined in Figure 3, which illustrates the conceptual framework for the controller’s augmentation or dosage recommendations, operating similarly to a finite state machine. Each state is represented by 2 digits: the first digit denotes augmentation PA (PA dosage) and the second digit indicates augmentation EI (healthy eating dosage), and either the PA dosage or EI dosage is updated at any given time. The controller recommends dosages with a minimum user-specified delay of 2 weeks between all dosages, except between dosage 2 and dosage 3, where a 3-week delay is specified. Transitions between dosages are governed by an auxiliary signal *a_k_*. If *a_k_* equals 1, the controller can transition from the current dosage state to the next; otherwise, it remains in the current dosage state.

### Goal of This Study

The goal of this study is to describe the protocol for a randomized controlled optimization trial to examine the efficacy of the enhanced HMZ 2.0 intervention with the new automated control system and digital platform to regulate GWG and influence secondary maternal and infant outcomes while collecting implementation data to inform future scalability. Aim 1 is to examine the efficacy of the intervention in terms of GWG (primary outcome) and maternal PA and EI behaviors and social cognitive determinants on comparing intervention and control groups. It is hypothesized that the intervention group will (1) have lower pre- to postintervention GWG and be more likely than controls to achieve GWG within the guidelines [1,2], and (2) have higher PA kcal (and total kcal) and PA or EI determinants and lower EI kcal than controls. Aim 2a is to measure pre- to postintervention differences in secondary maternal sleep and eating behaviors. It is hypothesized that the intervention group will have fewer nighttime awakenings and less uncontrolled eating than controls. Aim 2b is to examine the impact of the intervention on birth weight and adverse pregnancy, labor, and delivery outcomes. It is hypothesized that the intervention group will show lower birth weight adjusted for gestational age and will have lower occurrences of adverse pregnancy, labor, and delivery outcomes than controls. Aim 3 is to examine the impact of implementation markers on intervention efficacy in terms of GWG and secondary outcomes. It is hypothesized that subject engagement, acceptability, dosage exposure, and staff burden or acceptability will moderate the effect of the intervention on study outcomes. This information will inform how to scale-up the HMZ 2.0 intervention for future use by prenatal clinicians.

## Methods

### HMZ 2.0 Intervention Description

HMZ 2.0 is a multidosage, individually-tailored, adaptive intervention with social cognitive theory and behavior components [15-17]. The “baseline dosage” is delivered to all intervention participants, and it consists of up to 24 weekly modules (depending on gestational age at study enrollment) including the following:

- Education: Knowledge-based content on meeting guidelines (GWG, PA, EI, sleep behaviors, and good sleep hygiene) [1,2,69-75]; mood (eg, depressive symptoms, stress, and anxiety); safely engaging in prenatal exercise; and awareness of stressful situations that prompt uncontrolled and emotional eating, hunger cues, cravings, and mindful eating choices [76,77]. We also provide knowledge about recently published evidence-based studies on how a mother’s health impacts her baby (eg, sleep, brain development, and food preferences) and developmental milestones (eg, when eyebrows develop), which is shared over the course of the intervention. Our pilot work [42] demonstrated that women specifically asked for education materials that provided this information.
- Personalized behavior coaching or counseling: Individually tailored behavior coaching from a prenatal fitness instructor and registered dietitian on GWG, PA, and EI that uses information and feedback from a woman’s prior week to inform the future week’s motivational cues and strategies to increase PA, improve diet quality, and overcome barriers (this can be delivered in person or through a remote synchronous or asynchronous approach for future scalability).
- Goal setting and action planning: Guided and self-selected PA and EI goals [69-71,78] using implementation intentions to target when, where, and how each woman will work toward goals and how these PA or EI goals relate to GWG. Example goals include targets for daily steps (eg, 10,000 steps) and activity time (eg, 30 minutes), as well as fruit or vegetable intake for lower energy density [79] and increasing water intake to maintain good hydration [80,81]. HMZ trimester-specific prenatal PA and recipe booklets (developed by our team) provide detailed examples for PA (eg, 150 min/week of moderate-intensity activities; 10,000 steps/day) and EI kcal goals [78].
- Self-monitoring: Women use mHealth tools (eg, Wi-Fi weight scale, activity monitor, and dietary intake app) to self-monitor GWG, PA, or EI [82]. The HMZ digital platform visually displays their daily or weekly data. Feedback is given to each woman on how to use the devices and self-monitor their behaviors in relation to their GWG and PA or EI goals [19,43,78,83].

#### Adaptive Intervention Dosage Changes

**Figure 4:**
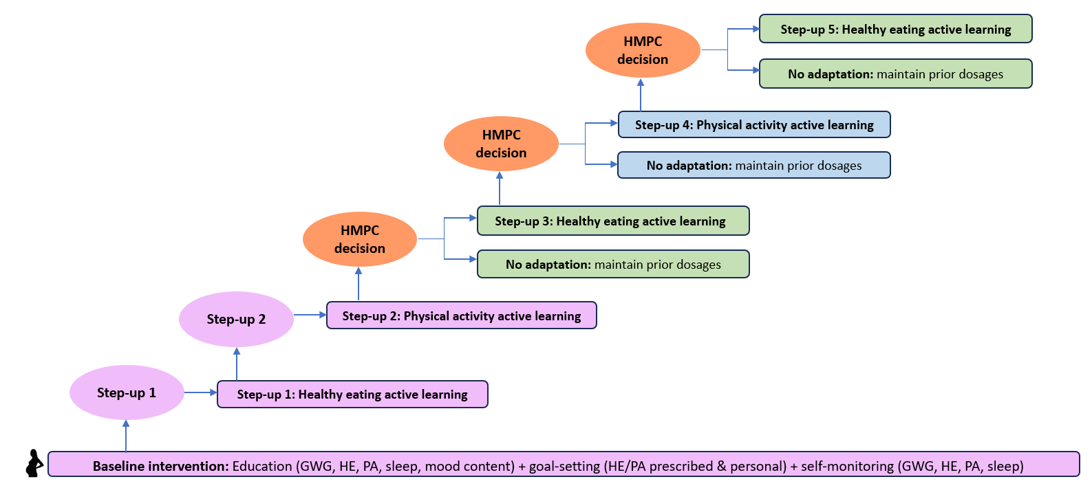
Healthy Mom Zone (HMZ) 2.0 adaptive intervention design. Participant weight is measured daily and continuously evaluated against the Institutes of Medicine (IOM) gestational weight gain (GWG) ranges. All intervention women will receive the baseline intervention plus step-up 1 plus step-up 2 during the system identification open-loop experiment. Dosages 3 to 5 are delivered to selected intervention participants based on hybrid model predictive controller (HMPC) decisions in the closed-loop experiment. HE: healthy eating; PA: physical activity.

The model-based predictive control algorithm in the HMZ 2.0 digital platform continually and automatically evaluates GWG. It relies on a dynamic model and solving a receding horizon online optimization problem that identifies when a woman’s forecasted GWG is anticipated to exceed the GWG guidelines [1,2,84-86], and based on a structured sequence of decisions that form part of the model, a dosage change is recommended (Figure 4). Only women who have a forecasted need for added support to regulate GWG will receive adaptive dosages in addition to the baseline intervention. Dosages are additive such that a woman receives more intensive support for eating healthy and engaging in PA with each adaptation up to a maximum of 5 adaptive dosages. The web-based user interface provides an easy and intuitive way to integrate expert-supervised dosage change recommendations to regulate GWG. The HMZ 2.0 prenatal fitness instructor and registered dietitian review the participant’s data and recommend individually tailored behavioral strategies that are delivered through the HMZ 2.0 digital platform. Each woman’s unique preferences and past successes with PA or EI strategies are considered when customizing the dosage adaptation to promote engagement, enjoyment, and compliance, which can in turn influence GWG. These active learning strategies include, for example, multiple PA-guided workouts with a variety of prenatal cardiovascular and resistance training exercises (preapproved for safety and with physician consent for participation) [69,70], as well as healthy eating cooking demonstrations, portion size control strategies to substitute high energy density foods with low energy density options such as water-rich fruits or vegetables [43,71], using food scales and portion size containers, customized grocery planning, and meal replacements. There are a host of easy-to-adopt and practical recommendations for integrating PA or EI strategies into daily life (eg, walking in 5- to 10-minute increments throughout the day to increase PA kcal by 200 kcal/day, reduce sitting by 5 min/hour from 9 AM to 5 PM, and replace 8 ounces of whole milk with skim milk to reduce EI kcal by 100 kcal/day) to facilitate behavior change while adhering to safety standards [69,70]. All dosages can be delivered in person or through a remote approach (synchronous and asynchronous for selected sessions), depending on the participant’s preference.

#### HMZ 2.0 Automated Data Pipeline and Digital Platform

**Figure 5:**
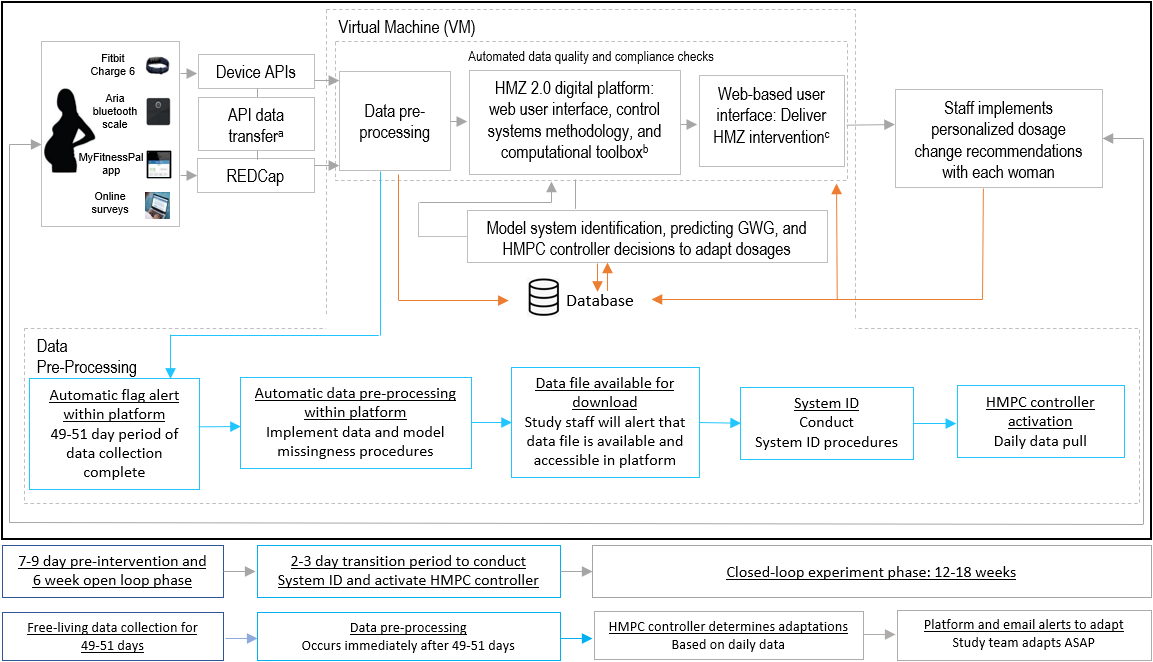
Healthy Mom Zone (HMZ) 2.0 data transfer pipeline. API: application programming interface; GWG: gestational weight gain; HMPC: hybrid model predictive controller. *Real-time deidentified 24-hour data stream into REDCap, **Access control (user ID) by authorized study staff, ***Access control (user ID) by intervention study staff (and in the future, dissemination to clinicians).

**Figure 6:**
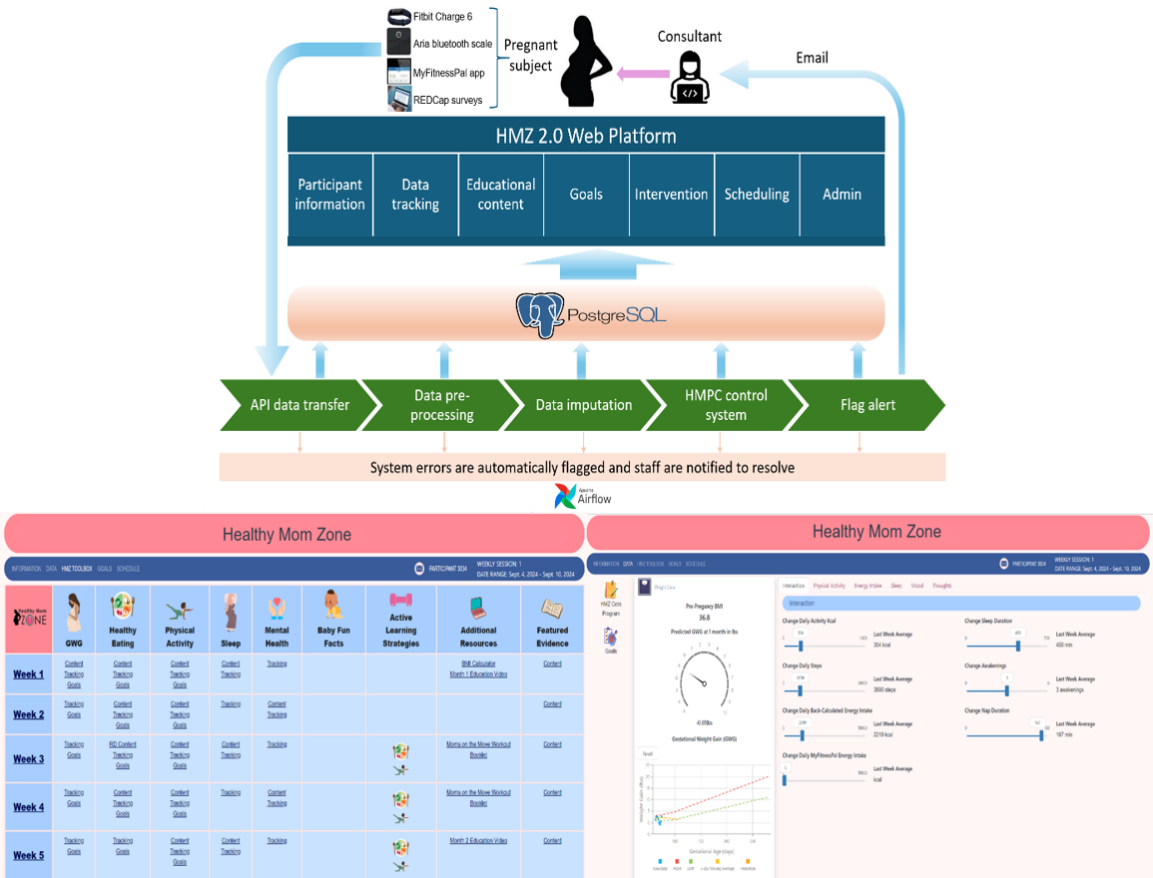
Healthy Mom Zone (HMZ) 2.0 digital platform architecture and website examples. API: application programming interface; HMPC: hybrid model predictive controller.

The data pipeline for HMZ 2.0, illustrated in Figure 5, is orchestrated by Apache Airflow [87], a workflow management platform, where daily jobs are scheduled, including (1) retrieving deidentified data from various sources, such as the application programming interface (API) of the REDCap [88] database; (2) preprocessing survey data, including aggregating subscales and back-calculating EI [89,90]; (3) imputing missing data via multiple imputation [91,92] and machine learning–facilitated [93-95] techniques programmed into the platform; (4) feeding the imputed data into the advanced hybrid model predictive control system to generate the adaptive decision-making process; and (5) continuously monitoring errors and warnings in the Airflow monitoring dashboard during steps 1-4, scanning data quality, and initiating automated alerts that notify different pillars of the study team based on the issue type. As the participants in the study cohort change with time, tasks for each new participant are dynamically created. The scripts are version controlled, and data generated throughout the pipeline are saved in a PostgreSQL relational database to ensure data reproducibility. The HMZ 2.0 web-based platform has been built with Django, an advanced Python web framework to scale-up future production and access (see Figure 6 for the digital platform architecture and website examples). Heterogeneous user interfaces have been built for study staff (and future clinicians), participants in the intervention group, and participants in the control group. The web-based platform provides an access-controlled admin dashboard where authorized users can manually update settings, curate data, or export deidentified datasets for further analyses. For example, during intervention sessions, study staff can access participants’ general information, data, educational content, goals, and intervention dosages, and participants will have access to selected resources that are tailored to their specific needs.

### Recruitment

Recruitment began in April 2024 and will continue through May 2027. Pregnant women with overweight or obesity (N=144) are being recruited for this study. Our team has extensive expertise with recruiting pregnant women. Our past studies have yielded over 3100 subject contacts, with recruitment rates of 84%-92% across our studies [19,96-102]. Well-established methods from our past studies are used for recruitment procedures as follows:

- Clinic: Nurses identify eligible women at the 1st prenatal visit (eg, appointment schedule and electronic health record) and refer them to the study team for screening. A study flyer with study contact information and a QR code is included in the clinic’s prenatal packet and posted in exam rooms.
- Community: Study flyers are posted in local areas (eg, daycares and churches) and hospitals, and shared at community events (eg, farmer’s markets and celebratory events).
- Social media: Study information is shared on Facebook, Instagram, X, YouTube, and study websites.

Using well-established procedures from our past studies [19,96-102], interested participants, regardless of recruitment method, are screened for eligibility. If interested, participants scan a QR code that directs them to a REDCap survey to complete a screening questionnaire or they send a message by email, text, or voicemail, and a trained study staff member will reply to complete the screening questionnaire over the phone.

The staff member reviews the participant’s responses to determine eligibility. The inclusion criteria are as follows: age range 18-45 years; singleton pregnancy with ≥8 and <18 weeks gestation; any parity; any race or ethnicity; BMI of 24-45 kg/m^2^ (>40 with provider consent); have not gained >25% of total GWG (based on BMI and IOM guidelines) from prepregnancy to the date of enrollment [103]; able to read or understand English; access to a computer or phone; able to attend sessions either on-site or remotely; randomization to conditions; no absolute contraindications to PA (and presence of relative contraindications only with health care provider consent to participate); and not a current heavy smoker (>20 cigarettes per day) [19,69-71] . The exclusion criteria are as follows: outside the age, BMI, gestation, or GWG range; not able to participate (cannot read or understand English, no access to a phone or computer to attend sessions remotely, and cannot use a device or service assistance); absolute contraindications to PA [69-71] or relative contraindications to PA noted by the participant’s health care provider as precluding study participation; current heavy smoker (>20 cigarettes per day) at study entry; and multiple pregnancy. Eligible women are scheduled for the preintervention assessment. Women not meeting the eligibility criteria are thanked for their time and given information on other studies that may be of interest.

### Safety Considerations

Prior to enrollment, a study team member informs the participant’s health care provider about their potential participation and obtains consent from each provider. The provider completes the consent form (hard copy or REDCap link) confirming eligibility for participation, and the participant is officially enrolled. This process is repeated at mid-study (eg, between the 2nd and 3rd trimesters) to ensure the participant does not have any new medical conditions that may impact study participation.

Because this study includes pregnant women, a vulnerable population as defined by the National Institutes of Health, there is a Data Safety and Monitoring Board with experts in obstetrics and gynecology, prenatal weight gain, PA and nutrition interventions, and fetal growth that will: (1) review the study methodology and procedures, data on recruitment, enrollment and adherence to the inclusion/exclusion criteria, and participant’s progress through the study; (2) assure the safety of the study participants; and (3) make recommendations to the research team. Adverse events that the Data Safety and Monitoring Board will be notified of and oversee include: (1) insufficient GWG: indicators of insufficient GWG are weight loss of less than 3% in a week or 0% weight gain in a 4-week cycle [104]; (2) depressive symptomology: all women regardless of initial preintervention assessment scores will be given resources on managing depressive symptoms and a comprehensive list of available resources and supportive services, and depressive symptoms will be monitored monthly [105]; and (3) absolute or relative contraindications to exercise [69-71].

Participants in the intervention group complete verbal assessments of pregnancy symptoms (eg, mild muscle cramping, headaches, and symptoms of labor) and contraindications to exercise (eg, bleeding, severe abdominal cramping, nausea, etc) during activity sessions. The study safety protocol includes steps to understand symptoms, ratings of perceived exertion, and responses if appropriate, including stopping the activity, seeking medical attention, and calling a participant’s emergency contact and provider. If a woman experiences a contraindication to PA in pregnancy that precludes her continued participation, she will remain in the study and complete measures as appropriate (intent to treat) but will not engage in PA until provider consent to return to activity is obtained. We also monitor changes in health status and health symptoms. For example, women who develop gestational diabetes during the study will remain in the study and receive the standard of prenatal care by their obstetrician or health care provider, in which an established standard of care plan is provided for the treatment and management of gestational diabetes.

### Ethical Considerations

This study has been approved by the Pennsylvania State University Institutional Review Board (IRB; STUDY00019075), and Arizona State University is an IRB-approved participating site (SITE00001437). All members of the study team have appropriate CITI training certifications. Any and all changes made to the protocol will be communicated to participants and other relevant individuals or parties immediately. All study participants provide their informed consent prior to enrollment into the study with the option to decline enrollment or stop participation at any time. Data collected are deidentified, and access is only granted through lock and key as well as secure accounts with passwords. Participants are compensated up to US $250 in gift cards for either Target or Walmart. Compensation is provided based on the completion of study milestones, such as completing the pre- and postintervention assessments, allowing electronic health record data to be extracted, and attending and completing more than 85% of study sessions and the measurement protocol. Both the intervention and control group participants can receive the same amount of compensation. This study is registered at ClinicalTrials.gov (NCT05807594).

### Randomization Procedures

A trained staff member randomizes each participant after the preintervention measures via a randomization module in REDCap [88]. The study’s biostatistician has developed the randomization scheme using variable-size random permuted blocks to ensure the number of subjects in each group is balanced after each set of *B* randomized subjects, where *B* is block size. The biostatistician programs the REDCap module, and the remaining study staff do not have access to the randomization scheme. Randomization to control (n=72) and intervention (n=72) groups uses 1:1 allocation; subjects are entered consecutively. Randomization is stratified by prepregnancy BMI status (<29.9 vs ≥30 kg/m^2^). A staff member calls each woman to inform her of study assignment. She is then provided with study information and education materials for her group assignment. The investigator is blinded to intervention assignments.

### Treatment Conditions

#### Intervention Condition

Women randomized to the intervention group receive the HMZ 2.0 intervention, described in detail above in the section “HMZ 2.0 Intervention Description,” which includes the baseline intervention (education, personalized behavior coaching or counseling, goal setting or action planning, and self-monitoring) and adaptive intervention dosage changes (for the participants who need them). This baseline intervention continues through the duration of the study. Each week over the course of the study, GWG is monitored and evaluated against the recommended upper and lower bounds of the IOM GWG guidelines [1]. Depending on each participant’s response to the baseline intervention (eg, GWG within or above guidelines) and her forecasted GWG, the automated control system may recommend a dosage change to adapt the intervention. This adaptive dosage begins after the first 4 weeks of the baseline intervention (to allow the participants to get used to the intervention and reduce burden) and includes EI and PA active learning interactive components that “step-up” the intensity of the dosage. Dosage change recommendations are automatically delivered through the HMZ 2.0 digital platform (architecture of the platform is described above); staff implement the recommended dosage change with the participant. As illustrated in Figure 6, there are up to five dosage “step-up” adaptations that can be recommended by the automated control system to regulate GWG.

#### Attention Control Condition

Consistent with guidelines for comparator groups [106], all women in the study receive prenatal care offered by recruitment sites with routine provider visits, counseling about prenatal behaviors (eg, no smoking), and clinical oversight of health. To match attention to the intervention group, women in the control group receive (for the first 4 weeks of the study) one-on-one weekly education sessions delivered by trained study staff, and thereafter, they receive monthly education content delivered as asynchronous videos and get check-in support from study staff (eg, phone, text, and email) for the remainder of the study. Content includes topics, such as preparing for labor or delivery; benefits of behavioral pain management strategies (eg, mindfulness-based relaxation, imagery, music, massage, and deep breathing) to regulate pain after childbirth with nonpharmacological approaches [107]; and baby or child safety, health and development, behaviors, and nutrition. Content is drawn from evidence-based guidelines and materials designed by members of the study team for a patient-provider toolbox to reduce opioid pain management use after childbirth [107], as well as The American Academy of Pediatrics, The March of Dimes, The Centers for Disease Control, and The American College of Obstetrics and Gynecology [108-111]. The matched control group education content is also provided to the intervention group as supplemental material.

Both the intervention and control groups receive the same measurement protocol and frequency of measurement to understand and compare the impact of the intervention on primary and secondary study outcomes. Data are collected from each participant daily (GWG: Aria Wi-Fi scale [112]; PA, sedentary behavior, or sleep: Fitbit Charge 6 monitor [112], logs for device wear time, and PROMIS Sleep Disturbance [113,114] assessment; and self-reported hydration behaviors); weekly (self-reported online in REDCap; social cognitive determinants and EI diet quality: MyFitnessPal app on 2 weekdays and 2 weekend days) [19,115]; and monthly (self-reported online in REDCap; eating behaviors [19,116,117], psychosocial measures, cognition, pain, and temperament measures).

### Control Optimization Trial Procedures

Control systems methodology [51-54] in a novel Control Optimization Randomized Controlled Trial (RCT) [51] is used to test the efficacy of the enhanced HMZ 2.0 intervention with a novel digital platform and a new automated control system to regulate GWG and influence secondary outcomes. This idiographic approach uses individual dynamic models (integrating behavioral and energy balance models) informing how each woman responds to HMZ 2.0 to make personalized decisions about intensifying dosages to regulate GWG. We are not aware of any other studies using this unique strategy to regulate GWG. Women start the intervention with an “open-loop” experimentation phase and receive the HMZ 2.0 baseline intervention (dosages are not yet adapted during this time). Semiphysical models estimated using concepts from system identification [85,118-120] allow individual energy balance and behavior models to be built for each subject [51]. Model and dosage personalization is further enhanced by leveraging our team’s expertise in time-varying dynamic systems (systems that show changes in statistical properties over time) and multilevel models that integrate individual- and group-based dynamics and missing data issues [56-68]. Model components are measured with real-time data procedures to predict corresponding deviations from each woman’s target GWG range: overweight, 6.8-11.3 kg total and 0.23-0.32 kg/wk; obese, 5.0-9.1 kg total and 0.18-0.27 kg/wk [1,2]. Model estimation is used to confirm individual models and identify if certain constructs provide maximum impact for the control system. The goal of this open-loop phase is to arrive at a set of personalized dynamic models that capture the effects of dosage augmentations, gestational age, and the baseline intervention. Once the models are identified and the controller commissioned, models will not be updated, but the tuning parameters can be adjusted, if necessary. While there is no set threshold of data compliance for building individual models (models can be built with as little as 50% missing data handled with maximum likelihood estimation) [56-68], we will rely on strategies to achieve high compliance (90%; 10% missing data) similar to that in our feasibility study [19].

These personalized individual energy balance and behavior models are used in the “closed-loop” experimentation phase for the rest of the study period during which a woman’s responsivity to HMZ 2.0 is considered and dosages are adapted to regulate GWG. Continuous mHealth data are automatically linked to the control system, which considers GWG over a prediction horizon to minimize discrepancies between a woman’s observed GWG and her goal [1,2]. It considers the anticipated rate of GWG change as predicted with the individual model. Currently trained with data from the feasibility trial [19] and pilot data collected on HMZ 2.0 participants, the control system uses the values of each woman’s modifiable factors (eg, PA or EI kcals) in the current week to simulate ways in which these behaviors can be “controlled” or altered to drive GWG closer to the goal in the following week. We are cognizant of potential system identification issues that may arise with limited data available from each subject. If needed, selected parameters from the energy balance models can be constrained to be invariant across subjects to borrow strengths from other subjects and aid model estimation [121,122]. We will identify the optimal balance between control system performance (how well it can produce desired effects as efficiently as possible) and robustness (how well it can produce desired performance under disturbances and uncertainty; eg, poor compliance, change in responsiveness to dosages, and measurement variability) [10,51]. The controller has tuning parameters that allow adjustment of how fast or slow the control system makes recommendations to enable a judicious balance between effectiveness and responsiveness. Computations are performed with toolkits in MATLAB (MathWorks) and IBM ILOG CPLEX Optimizer. Study staff work with subjects to address concerns or technical issues. We will also explore how the individual subject data may inform modifications to the maternal energy balance and behavior model [10,19] and infant birth weight model [11,18].

### Primary and Secondary Outcomes

Table 1 summarizes the HMZ 2.0 measurement protocol and timepoints. More details of the primary and secondary outcomes are provided below.

**Table 1:** Healthy Mom Zone 2.0 measurement protocol and timepoints.

Variable measure				Timepoint
**Energy balance model outcomes**				
	**GWG^a^** **/weight (primary outcome)**			
		High precision adult scale (10 s)	Pre- and postintervention	
		Aria Wi-Fi Smart Scale (15 s)	Pre- and postintervention and daily	
		Prenatal records: total GWG	Pre- and postintervention	
	**PA^b^** **and sedentary behavior**			
		Fitbit Charge 6: activity kcal (passive)	Pre- and postintervention and daily	
		ActiGraph GT3X: activity min (passive)	Pre- and postintervention and daily	
		PA log: monitor wear time (1 min)	Pre- and postintervention and daily	
	**Resting metabolic rate**			
		Predicted equation	Pre- and postintervention and daily	
	**EI^c^**			
		EI kcal: back-calculation method	Pre- and postintervention and daily	
		EI diet quality: MyFitnessPal app (5 min)	Pre- and postintervention and monthly	
	**PA or EI social cognitive determinants**			
		Attitude, subjective norm, perceived behavioral control, and intention (3 min)	Pre- and postintervention, daily, and weekly	
		Behavioral, normative, or control beliefs (2 min)	Pre- and postintervention and monthly	
		Retrospective self-regulation (1 min)	Pre- and postintervention, daily, and weekly	
**Secondary outcome measures**				
	**Sleep behaviors**			
		Pittsburgh sleep quality index (2 min)	Pre- and postintervention and monthly	
		FitBit Charge 6: sleep behaviors (passive)	Pre- and postintervention and daily	
		Sleep log: sleep behaviors (30 s)	Pre- and postintervention and daily	
		PROMIS Sleep Disturbance (30 s)	Pre- and postintervention and daily	
	**Eating behaviors**			
		3-factor eating inventory: cognitive restraint, disinhibition, and hunger (3 min)	Pre- and postintervention, weekly, and monthly	
	**Maternal-infant labor or delivery and adverse pregnancy outcomes**			
		Diagnoses of gestational diabetes, insulin use, preeclampsia, and depression	Postintervention only (at delivery)	
		Labor or delivery issues and other complications	Postintervention only (at delivery)	
		Infant APGAR score and mode of delivery (vaginal or cesarean)	Postintervention only (at delivery)	
	**Infant birth outcomes**			
		Birth weight (adjusted for gestational age at delivery), length, sex, gestational age at delivery, and date of birth in the electronic health record	Postintervention only (at delivery)	
**Clinical and safety protocol measures**				
	**Height**			
		Stadiometer (5 s)	Preintervention only	
	**Blood pressure**			
		Screen for preeclampsia (2 min)	Preintervention only	
	**Demographics, medical history, and obstetric history**			
		Age, race or ethnicity, income, education, medical or pregnancy history, etc (7 min)	Pre- and postintervention	
	**Depressive symptoms and monitoring health**			
		Center for Epidemiological Studies Depression Scale (3 min)	Pre- and postintervention and monthly	

^a^GWG: gestational weight gain.

^b^PA: physical activity.

^c^EI: energy intake.

### Primary Outcome

#### Participant GWG

Weight and GWG are assessed daily at preintervention, during the intervention, and at postintervention at home using the Fitbit Aria Wi-Fi Smart Scale [112] (weights are wirelessly uploaded to an online program). GWG is standardized, and the target weight gain is determined for each woman based on the BMI status (overweight, 14.1-22.7 kg; obese, 11.3-19.1 kg) [1,2]. For the criterion measure to determine when to adapt the intervention, weight gain is calculated to determine if a woman is gaining less than her goal, at the exact amount of her goal, or more than her goal. GWG over the course of the study is calculated as the last measured weight during the study subtracted by the first measured weight during the study. Weight at enrollment is measured, and prepregnancy weight and GWG from the first prenatal visit to the last predelivery weight are abstracted from clinical records.

#### Energy Balance and Behavior Model Primary Constructs

##### PA and Sedentary Behavior

PA and sedentary behavior are assessed daily at preintervention, during the intervention, and at postintervention. Participants wear the wrist-worn Fitbit Charge 6 [112] 24 hours per day from the preintervention assessment until the end of the postintervention assessment. The Fitbit Charge 6 allows for continuous passive (low subject burden) PA assessment in the energy balance model [12] to predict GWG. The device measures total kcal, activity kcal, steps, and minutes in sedentary, light, or moderate PA. The waist-mounted ActiGraph GT3X [123] is worn at pre- and postintervention and for the first 2 weeks of the open-loop phase during waking hours to assess PA and sedentary behavior (activity kcal, steps, and minutes in sedentary, light, or moderate PA). Participants track their PA and monitor wear time by completing a self-report PA log and the Leisure Time Exercise Questionnaire [124] daily for cross-validation of the Fitbit data [125].

##### EI Behavior: Back-Calculation Estimation

EI is estimated daily at preintervention, during the intervention, and at postintervention from measured weight (Aria Wi-Fi scale) [112], PA (Fitbit Charge 6 activity monitor) [112], and resting metabolic rate (RMR), with k=1, 2,… N relating to day 1 to day N. T is the sampling time (T=1 day) [8,12,68-71], and RMR is estimated daily as follows:

eRMR = 0.1976W^2^ – 13.424W + 1457.6 **(1)**

The noise in weight is small relative to the total weight, but the extent of this noise can affect the calculated rate of GWG per day, so a 5-day moving average filter is used to preprocess (smooth) measured weight before “true” daily EI is estimated [117-119]:



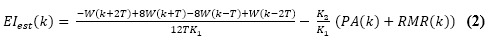



Our team effectively used this back-calculation method for estimating EI in the HMZ proof-of-concept study [42] and feasibility-initial impact randomized trial [8,10,19,43-45].

### Secondary Outcomes

#### Secondary Energy Balance and Behavior Model Constructs

The following aspects are considered:

- Theory of Planned Behavior [15,126]: Involves healthy eating or limiting unhealthy eating, PA attitude, subjective norm, perceived behavioral control, intention, and beliefs. The Theory of Planned Behavior constructs in the dynamic model of energy balance and behavior [13-17] inform individualized model-based interventions for each intervention participant. Participants complete online surveys daily at pre- and postintervention and during the open-loop phase, and daily and weekly during the intervention to assess their attitude, perceived behavioral control, subjective norm, intention to eat healthy and limit unhealthy eating, and participation in PA. Participants complete a one-time online survey at pre- and postintervention and monthly to assess their beliefs about eating healthy, limiting unhealthy eating, and participating in PA.
- Retrospective self-regulation for EI or healthy eating and PA [17]: Retrospective self-regulation in the dynamic model of energy balance and behavior [13-17] informs individualized model-based interventions for each intervention participant. Participants complete two 6-item online surveys daily at pre- and postintervention and during the open-loop phase, and weekly during the intervention to determine how good they are at regulating their EI or healthy eating and PA and how these behaviors impact GWG over the course of pregnancy.

#### Maternal Health

Participants complete the following measures at preintervention, during the intervention, and at postintervention:

- Diet composition or quality and eating behaviors: Participants use the MyFitnessPal online app on 2 weekdays and 2 weekend days at pre- and postintervention, weekly during the first 2 weeks of the open-loop phase, and once a month during the intervention to assess their diet composition and quality. This information aids in personalized counseling of diet quality for the intervention group [19]. The Three Factor Eating Questionnaire [116,117] is completed at pre- and postintervention, weekly during the open-loop phase, and monthly during the closed-loop phase and is used to assess 3 dimensions of eating behaviors: dietary restraint (cognitive control of eating behavior), dietary disinhibition, and susceptibility to hunger.
- Sleep behaviors: The Pittsburgh Sleep Quality Index [127] assesses the quality and patterns of sleep and measures subjective sleep quality, sleep latency, sleep duration, habitual sleep efficiency, sleep disturbances, use of sleeping medication, and daytime dysfunction. The PROMIS Sleep Disturbance short-form survey [113] assesses difficulties and concerns with getting to sleep and staying asleep; evaluates the perceptions of the adequacy of and satisfaction with sleep; and measures sleep quality, sleep depth, and restoration associated with sleep. Participants also complete a daily self-report sleep log [128] to measure time to sleep and wake, minutes of sleep, nighttime awakenings, time spent awake after sleep onset, daytime naps, and daytime nap duration, and wear the Fitbit Charge 6 Activity Monitor [112] each night to assess time to sleep and wake, minutes of sleep, nighttime awakenings, and light and deep rapid eye movement sleep.

#### Maternal and Infant Outcomes

Prenatal, labor, and delivery data will be abstracted from the participant’s electronic medical record. Maternal and infant outcomes include: mode of delivery (vaginal or cesarean), adverse pregnancy outcomes (eg, diagnosis of gestational diabetes, insulin use, preeclampsia, depression, labor or delivery issues, and other complications), birth weight, length, sex, gestational age, date of birth, APGAR score, and any complications related to the infant during labor and delivery. In the event that labor or delivery data are missing or unavailable from the medical record, the investigators will obtain information from the participant’s self-report.

### Implementation Marker Procedures

Our team has ample experience with evaluating program implementation [96-98,129-133], including in the HMZ proof-of-concept study [42] and feasibility trial [19]. The Quality Implementation Framework [49] and Quality Implementation Tool [50] will guide examination of the following markers:

- Subject engagement and participation: Fidelity monitoring evaluations are conducted for each subject and staff after each session. A trained staff observer reviews video recordings of 50% of intervention and attention control sessions and follows a review checklist to measure engagement (degree of subject responsiveness with content, discussion, and activities) [134,135].
- Subject acceptability: Subjects and staff complete weekly checklists to assess attendance (0%-100% attendance at pre- and postintervention assessments; 0%-100% attendance at intervention and attention control sessions), compliance (0%-100% compliance with mHealth tools and surveys during free-living pre- and postintervention sessions and over the course of the study for all subjects; 0%-100% compliance with attention control or HMZ 2.0 activities), and quality and completeness of data (0%-100% of usable data for each subject).
- Dosage exposure is assessed by (1) weekly checklists completed by subjects or staff regarding the amount of program content delivered or received (goal 85%+ coverage) [19]; (2) fidelity monitoring by a trained staff observer who reviews video recordings from the same sample of 50% of video recordings noted above; and (3) tracking of the number of dosage changes determined by the model-based control system, number of days between dosage change recommendation and implementation by staff, and number and type of PA or EI strategies suggested or used.
- Staff engagement: Fidelity monitoring is conducted by a trained staff observer who reviews video recordings from the same sample of 50% of video recordings noted above and follows a checklist to measure engagement (enthusiasm, preparedness, session delivery effectiveness, and responsiveness to the subject).
- Staff burden: Weekly checklists are completed by staff for the amount of time spent preparing for sessions, responding to or following up with subjects, and using the HMZ 2.0 digital platform, and for issues with delivering dosage recommendations to the subjects.
- Study delivery costs: A trained staff member tracks and calculates the time and financial costs of the study. Postintervention semistructured interviews with HMZ 2.0 subjects elicit key facilitators and barriers to study participation. The interview guide has been developed with standard procedures [136,137] from our past studies [10,19,42-45,107,138-140]. Expert feedback will be obtained from key clinician stakeholders (ie, registered dietitians, nurses, obstetrics or gynecology providers, etc) on the strengths and limitations of using the HMZ 2.0 digital platform, potential barriers to scaling-up use in prenatal care (eg, clinician beliefs about technology and connectivity issues), and suggestions for improvements. This valuable feedback will be used to make additional refinements to HMZ 2.0 to scale-up future use by prenatal care clinicians.

### Statistical Analysis

For study aims 1 and 2, time series methodology will be used on a subject basis (ie, cubic polynomial spline 3-lag auto-regressive models with maximum likelihood as the estimation method) followed by a fixed effects meta-analytic approach to combine information in order to compare the intervention group to the control group [141-143]. Mixed effects models will be used when the meta-analytic approach does not fit the data [56,62-65]. Potential confounding variables (eg, maternal age, parity, and income; infant sex; and adverse maternal-infant outcomes) [6,144] will be included in the models as covariates when appropriate. We will explore the extent to which prenatal sleep and eating behaviors impact postintervention GWG, PA, or EI and infant birth weight to inform modifications to the maternal energy balance and infant birth weight models. The intent-to-treat principle will be followed. Our statistical approach is robust if data are missing at random (multiple imputation will be considered if missing data are problematic) [145]. Hypothesis tests will invoke a 2-sided significance level of .05. Analyses for the synthesis of mHealth data and the predictive control system are informed by MATLAB toolkits and past experience [51,146,147]. Model personalization will use control-theory models, and requisite computations will be performed for optimal dosage changes [112,113,128,148-150]. For study aim 3, descriptive statistics will be used to examine the percentage and frequency for implementation markers. Formative methods will be used to organize, code, and rank order interview data [136,137]. Study findings will be used to further optimize HMZ 2.0 for effectiveness and scalability. Data will be collected and stored in REDCap [88]. Deidentified data will be exported from REDCap [88] to statistical packages. Subject confidentiality will be maintained with subject IDs. Data security is ensured with Penn State standard procedures.

### Power Calculations

The primary outcome is GWG: absolute difference between the intervention and control groups for the mean change in GWG from the start to the end of the trial. From our feasibility study [19], we found that the difference between the intervention and control groups with respect to the absolute mean change in GWG from the start to the end of the trial was 1.9 kg (SD 4.9 kg), with an approximately 21% relative reduction for the intervention group compared to the control group. We compared the mean change in our feasibility trial [19] to that in several other GWG studies comparing intervention and control groups [29,151-154] and found that the intervention group gained on average 1.8 kg less than controls (range: 1.1-3.1 kg). The sample size of 144 randomized to the intervention (n=72) and control (n=72) groups in the proposed research is informed by our feasibility trial showing a mean difference of 1.9 kg (SD 4.9 kg), a 21% relative reduction in GWG in the intervention group compared to the control group, and a 3% dropout. Given our experience with the feasibility trial [19], the series of refinements that we performed following the feasibility trial (eg, adding content on sleep and eating behaviors given their effects on GWG; modifying all content for remote delivery given 100% compliance found for remote sessions in the feasibility trial; replacing the initial control system to make dosage change decisions with the new, automated, dynamic model-based predictive control system that outperformed the initial system for regulating GWG; and adding the novel HMZ 2.0 digital platform to adapt dosages), and the findings from our HMZ 2.0 pilot study (average weekly GWG over the 4-week study period: 0.3 kg, SD 0.4 kg; overall GWG from pre- to postintervention: 1.2 kg, SD 0.8 kg), we expect the HMZ 2.0 intervention to be more effective. We anticipate that our mean difference will be closer to what a recent study [155] found for the mean difference in GWG between the intervention and control groups (mean 3.6 kg, SD 5.7 kg). Conservatively, considering this larger SD of 5.7 kg and anticipating a larger dropout rate of 10%, the sample size of 144 yields 80% statistical power with a 2-sided significance level of .05 to detect an absolute difference of at least 2.9 kg in GWG between groups. Sample sizes for semistructured interviews are adequate to observe data saturation [136,137,141].

## Results

### HMZ 2.0 Control Optimization Efficacy RCT

**Figure 7:**
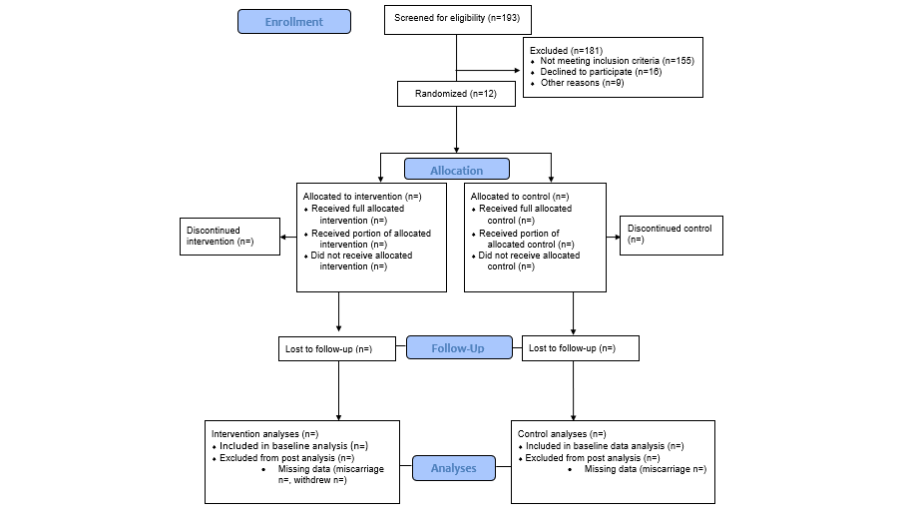
Healthy Mom Zone (HMZ) 2.0 CONSORT (Consolidated Standards of Reporting Trials) diagram.

Recruitment began in April 2024 and will continue through May 2027. All data are expected to be collected by December 2027. Currently, we have 193 participant contacts, of which 119 (91%) have been assessed for eligibility and 12 (50% of eligible participants) are enrolled in the RCT (Figure 7). Full results will be uploaded on the ClinicalTrials.gov website at the end of the trial, which is anticipated in January 2028.

## Discussion

### Overview

To our knowledge, there are no other adaptive behavioral interventions aiming to regulate GWG with a theory-driven, energy balance model–based predictive control system, highlighting the novelty of our approach. We have made every effort to obtain robust and unbiased results in this trial. We have established the proof-of-concept and tested the feasibility of the intervention dosages, safety protocols, and strategies to retain subjects for this trial. The hypotheses to be tested are based on a sound foundation of preliminary data [10,11,13,14,19,42-45,140]. We have also pilot tested several aspects of HMZ 2.0. Findings from the HMZ 2.0 pilot study showed successful recruitment from multiple methods, excellent participant compliance with the measurement protocol and transfer of data from devices and online surveys to the digital platform, user acceptability of intervention sessions delivered through the platform, and utility of the predictive controller for informing dosage change decisions. We will use a randomized study design for the control optimization trial to test the efficacy of the HMZ 2.0 intervention and automated data pipeline and digital web-based platform compared to an attention control group. Data will be collected from all subjects to measure primary and secondary outcomes with valid and reliable measures. We will show the reproducibility of mHealth measures by having all subjects complete the same measures over time. Intervention content, measurement and implementation evaluation protocols, and intervention materials are available for reproducibility. The HMZ 2.0 digital platform and new model-based predictive control system have been built in MATLAB Compiler for reproduction with limited license restrictions. The HMZ 2.0 digital platform is a central hub for the automated data pipeline and web interface for intervention delivery. The data pipeline uses Apache Airflow for robust and dynamic scheduling and implements a multilayer real-time monitoring system. The web interface leverages the Django framework to deliver a rich user experience with role-based access control. The digital platform greatly improves the efficiency of this collaboration study by facilitating information sharing and reducing the turnaround time among researchers, consultants, and patients. Since both Airflow and Django are readily scalable (eg, through parallelism and caching), this digital platform can easily accommodate scale-up in the future.

### Limitations

There are many strengths of this study, including clinical and public health significance, novel methods, introduction of the HMZ 2.0 digital platform and model-based predictive control system with personalized dynamic energy balance models for each subject to automate dosage changes and predict GWG, use of a back-calculation method to estimate EI, pilot data [10,11,13,14,19,42-45,140] to support the rigor and reproducibility of the proposed methods, and strong potential for future scalability. Despite these strengths, participant compliance in RCTs is an ongoing challenge, and thus, we have incorporated strategies (eg, staff support, easy passive data collection, and remote delivery of the intervention) to facilitate compliance. We have also worked through technical aspects of the data pipeline and digital platform to reduce technical challenges. Lastly, there is a risk that the HMZ 2.0 study may be underpowered based on the assumptions used in the sample size estimation, but this is true for any power analysis.

### Conclusions

The approach involving the HMZ 2.0 intervention and use of a control optimization trial [46] to examine the efficacy of the intervention in terms of GWG and related maternal-infant outcomes expands the boundaries of GWG interventions, uses novel methods and automated decision making, and has clinical and public health impacts in a high-risk population of pregnant women with overweight or obesity and their offspring. There is excellent potential to further refine HMZ 2.0 in the future to regulate GWG in women of all BMI categories and scale-up HMZ 2.0 for use by clinicians as an adjunct treatment in prenatal care. There may also be a way to optimize the digital platform in the future so that it can be safely and directly used by pregnant women. HMZ 2.0 may also serve as a model for how the control systems methodology and a digital platform can be used to automate dosage change decisions for other behavior interventions such as those involving regulation of diabetes, weight loss, and related health outcomes.

## Abbreviations

EI: energy intake
GWG: gestational weight gain
HMZ: Healthy Mom Zone
IOM: Institutes of Medicine
IRB: Institutional Review Board
PA: physical activity
RCT: randomized controlled trial
REDCap: Research Electronic Data Capture
RMR: resting metabolic rate

## Data Availability

Data cannot be shared publicly because the data collected from human subjects have not been approved for public sharing. Moreover, since the data are of human subjects, a data transfer agreement is needed. Data are available from the principal investigator DSD (dsd11@psu.edu or 814-863-0456) and the Pennsylvania State University Office for Research Protections (orp@psu.edu or 814-865-1775) for researchers who meet the criteria for access to confidential data.
